# Doping the Spin-Polarized Graphene Minicone on Ni(111)

**DOI:** 10.3390/nano14171448

**Published:** 2024-09-04

**Authors:** Cesare Tresca, Gianni Profeta, Federico Bisti

**Affiliations:** 1 CNR-SPIN c/o Dipartimento di Scienze Fisiche e Chimiche, Università dell’Aquila, Via Vetoio 10, 67100 L’Aquila, Italy; gianni.profeta@univaq.it; 2 Dipartimento di Scienze Fisiche e Chimiche, Università dell’Aquila, Via Vetoio 10, 67100 L’Aquila, Italy

**Keywords:** two-dimensional materials, theoretical modelling, magnetism

## Abstract

In the attempt to induce spin-polarized states in graphene (Gr), rare-earth deposition on Gr/Co(0001) has been demonstrated to be a successful strategy: the coupling of graphene with the cobalt substrate provides spin-polarized conical-shaped states (minicone) and the rare-earth deposition brings these states at the Fermi level. In this manuscript, we theoretically explore the feasibility of an analogue approach applied on Gr/Ni(111) doped with rare-earth ions by means of density functional theory calculations. Even if not well mentioned in the literature, this system owns a minicone, similar to the cobalt case. By testing different rare-earth ions, not only do we suggest which one can provide the required doping but we also explain the effect behind this proper charge transfer.

## 1. Introduction

The exceptional properties of graphene [1,2] (Gr) have stimulated studies in engineering Dirac bands, giving rise to the stabilization of new and interesting electronic phases. Exceptional examples include the superconducting phase [3,4], magnetic systems [5,6] for applications in spintronics, heterostructures showing new properties, twisted and Moiré configurations [7], nanostructures [8,9] and photonic applications in linear and non-linear optics [10,11], opening new routes for technological applications [12,13,14,15,16]. At the moment, the most promising ways to tailor graphene physical properties is by means of hetero-atom deposition, substrate-induced interaction-thought electronic and structural modifications, and twisted and/or heterostructures formed with 2D systems [17].

In particular, its growth or transfer onto different substrates has been the subject of significant investigations in the past, with the aim of understanding how its electronic properties change from the isolated picture (‘free-standing’) as a result of interaction with the substrate [15,18,19,20,21,22,23,24,25] or following atomic intercalation and doping [26,27,28]. In addition, the interface between graphene and different substrates was recently demonstrated to promote the growth of innovative two-dimensional graphitic-like systems [29]. The most important parameters defining the final graphene electronic properties are the lattice-matching and the degree of hybridization of π bands of graphene with the substrate. For example, the graphene grown on substrates with large lattice mismatch as Ir(0001) or Ru(0001) retains the linear π-bands’ dispersion close to the Fermi level without appreciable doping. On the contrary, lattice-matched Ni(111) and Co(0001) substrates strongly interact with graphene electrons while exhibiting spin-polarized bands [15,25,30,31,32]. In such strongly interacting scenarios, the peculiar high-electronic-charge mobility coming from the almost-linear dispersion of the Dirac bands could be compromised if they are completely destroyed by the interaction with the substrate.

This is not the case for Graphene growth on Co(0001). Indeed, even if a gap at the Dirac point of ∼0.4 eV is gained from the interaction with the substrate, the carbon π-bands are still highly dispersing bands (commonly called “minicone”) [30,33].

These features can result in the already known capability of graphene in relevant spintronics applications once they are combined to sustain spin currents after being injected by spin-polarized electrodes [34] if low spin-orbit coupling, negligible hyperfine interaction, and gate tunability are preserved. A fundamental element to consider is that the minicone in Gr/Co(0001) results in the lower part of split cones to be fully occupied and the upper part to be fully unoccupied, determining a null contribution in the electric conduction. Finding a process that may partially occupy these minicones is therefore necessary in order to take advantage of them. One such mechanism is the deposition of dopant adatoms, which increases the electron charge on the carbon layer. Although this procedure has been efficiently performed for quasi-free-standing graphene [26,27,28,33,35,36,37,38,39,40,41], in the present case, it is fundamental to avoid the intercalation of the dopant adatoms between the substrate and the graphene adlayer. In fact, intercalation tends to detach graphene from the substrate [31,40,42,43], destroying the spin-polarized states induced by the strong hybridization with the magnetic substrate.

Very recently, the low-temperature deposition of dopants, namely Europium adatoms on Gr/Co(0001), was demonstrated to be an effective technique to adsorb the dopants on a graphene sheet in an ordered $\sqrt{3}\times \sqrt{3}$-R30° reconstruction, without intercalation, and thus, heavily doping the minicone and keeping its peculiar spin-polarization [33]. This finding paves the way for the search of other magnetic substrates for the observation of an analogue mechanism.

A good candidate is Ni(111); the electronic band dispersion of graphene growth on Ni(111) shows the presence of the spin-polarized minicone [25,44,45] in analogy with graphene on Co(0001), guaranteeing a magnetic ordering above room temperature [40].

At the same time, if the intercalation is precluded (as by deposition at low temperature), RE adatoms are expected to reconstruct into an ordered surface on top of graphene transferring an electronic charge to it, as demonstrated in the case of Eu on Gr/Co(0001) [33].

The scope of the present manuscript is precisely to examine the doping mechanism induced by rare-earth (RE) deposition on the Gr/Ni(111) minicone using first-principles density functional theory (DFT) calculations. The considered RE (RE = La, Eu, Gd, Yb) adatoms are expected in a +2 configuration, and adsorbed on graphene in a $\sqrt{3}\times \sqrt{3}$-R30°. The chosen RE adatoms provide a comprehensive picture on the influence of the different adatoms’ electronic configuration on the minicone doping and dispersion, highlighting the relevant role of RE-*d*-states.

Finally, the calculations are expanded outside the domain of rare earth to illustrate that analogous mechanisms can also be traced back in Lu and Y, demonstrating the wider valence of the presented concepts.

## 2. Computational Methods

Theoretical calculations were performed using the Vienna ab initio simulation package (VASP v.6.4.2) [46], using the generalized gradient approximation in the revised Perdew–Burke–Ernzerhof version (PBEsol) [47] for the exchange-correlation energy. We used projected augmented-wave pseudopotentials [48] for all the atomic species involved, with an energy cutoff up to 500 eV. The surfaces were simulated within a supercell approach, which considers 6 Ni layers along the [111] direction and about 20 Å of vacuum, in agreement with the literature [25].

Graphene was adsorbed on the topmost Ni surface layer at the experimental lattice parameter 2.49 Å, in the 1×1 reconstruction with the top-fcc stacking. The ferromagnetic (FM) solution was considered for the Ni atoms in the calculations, while different spin-configurations were considered for the magnetic adatoms adsorbed on the hollow site of graphene [49] in a $\sqrt{3}\times \sqrt{3}$-R30° reconstruction in complete agreement with the Eu adsorption on Gr/Co(0001) [33]. The integration of charge density over the two-dimensional Brillouin zone (BZ) was performed using an uniform 6 × 6 Monkhorst-and-Pack grid [50] with a Gaussian smearing parameter σ=0.05 eV.

Total energy minimization was performed for all the atoms except for the bottom-most four Ni layers that were fixed to their Ni bulk positions. The DFT + U approximation was adopted for the treatment of the *f*-orbitals, with the U and J parameters chosen in agreement with the literature (for Eu and Gd, we adopted U = 5.9 eV, J = 0.9 eV [31]; for Yb, U = 2.0 eV, J = 0.7 eV [45]; while for La, Lu and Y, no correction is needed).

## 3. Results

We start our study with a graphene/Ni(111) system taken as a reference. The adsorption distance between graphene and the Ni surface is predicted to be 2.05 Å in agreement with previous studies [25,45]. Nickel substrate induces small magnetic moments on the carbon atoms, showing an anti-ferromagnetic order between nonequivalent carbon sites (carbon in the on-top position has a magnetic moment aligned with those of the Ni substrate (see Table 1).

Once the RE adatom is included in the calculation, its adsorption causes the increase in the graphene vertical distance from the Ni(111) substrate with respect to the undoped case, regardless of the RE atom involved (see Table 1). This effect is the natural consequence of the electronic charge transfer from the RE atom to graphene, as it will be shown in the electronic band structure (see below). The adsorption distance between RE atoms and the carbon layer is larger (of about 2.2 Å) for La and Gd than in the case of Eu and Yb (of about 2.1 Å).

The structural properties correlate with the magnetic interaction between RE and the substrate; the large (small) graphene–substrate distance of La and Gd (Eu and Yb) corresponds to their ferromagnetic (anti-ferromagnetic) arrangement with respect to the Ni substrate (even if Yb shows a very poor residual magnetization). Such ordering of the adatoms influences the magnetic moment of the last Ni layer at the surface, giving an enhancement (reduction) in the ferromagnetic (anti-ferromagnetic) configuration (see Table 1). In all the considered cases, the fragile magnetic ordering present in carbon atoms in the Gr/Ni(111) system is practically destroyed by the presence of RE adatoms due to the increased graphene–substrate distance.

**Figure 1:**
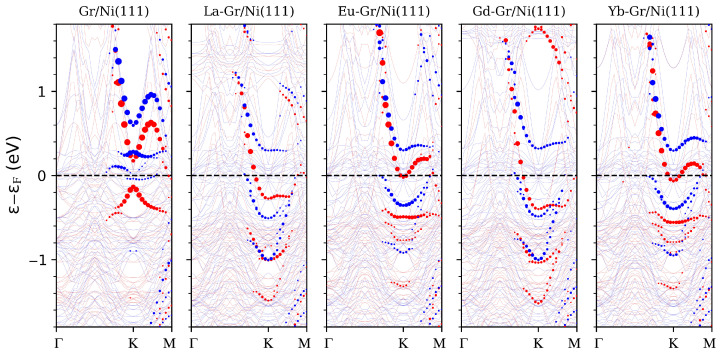
From left to right, with red (blue) lines representing the majority (minority) spin channel band structure of the pristine system followed by La, Eu, Gd and Yb adatoms on Gr/Ni(111). The band dispersions are along the Γ−K−M direction of the graphene 1 × 1 BZ. The size of red (blue) circles is proportional to the projection of the respective eigenfunction, from the majority (minority) spin channel, on the carbon $p_{z}$-states further unfolded on the graphene 1 × 1 periodicity.

The analysis of the electronic properties reveals the origin of these different behaviours. In Figure 1, we report the spin-polarized band structures for the considered systems, unfolded on the graphene BZ 1 × 1 cell and projected on the C-$p_{z}$ orbitals to facilitate the recognition of the most dispersive graphene bands.

In line with previous studies [25,45], both majority and minority spin components exhibit a gap opening at the Dirac point, resulting in the so-called “minicone” shape at the K-point. Only the majority spin component has occupied valence states, separated by an energy gap of ∼0.35 eV from the conduction valley (see red dots in Figure 1). The minicone gap opening originates from two main effects: the sublattice asymmetry induced by the Ni(111) substrate and the exchange field due to the strong p−d hybridization coming from the spin-split Ni *d*-orbitals.

The adsorption of RE atoms drastically changes the electronic properties of the system: it induces electron doping, shifting downwards the spin-polarized carbon bands, and it brings strong modification of the graphene minicone, producing an overall flattening of the band (in particular, along the K–M direction; see below).

Similarities in La and Gd behaviour opposed to the Eu and Yb cases are recognizable. First of all, the former provides a higher doping regime (downward shift of −0.6 eV) than the latter (−0.2 eV). Then, such higher doping present in La and Gd is accompanied by a strong flattening of the majority spin band dispersion along the K–M direction, placing it below the Fermi level at about −0.25 eV. Rather, instead, in the case of Eu and Yb, the graphene conduction majority spin band is almost unaltered in shape. All these effects can be connected to the valence configuration of the RE atom involved.

In fact, both La and Gd have *d* states in valence (electronic configurations are $\left[Xe\right]\;5d^{1}\;6s^{2}$ and $\left[Xe\right]\;4f^{7}\;5d^{1}\;6s^{2}$, respectively), and those states tend to bond with graphene $p_{z}$. A hybridization of the RE-$d_{xy/x^{2}-y^{2}}$ orbitals with the C-$p_{z}$ ones can completely disrupt the minicone structure, giving rise to a semi-parabolic dispersion along the ΓK path. In addition, the band becomes extremely flat from K to M and, via a super-exchange mechanism, the RE results in a magnetic moment aligned parallel to the Ni(111) substrate.

**Figure 2:**
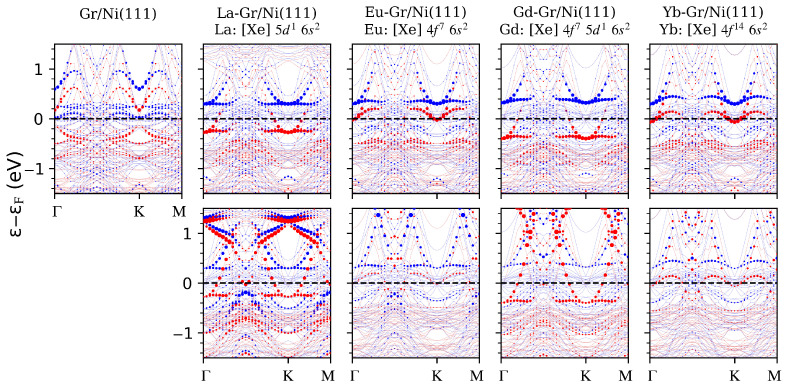
First row: electronic band structures for the studied systems projected on the surface-Ni($d_{z^{2}-r^{2}}$) states (**top**) and on the RE($d_{xy}+$$d_{x^{2}-y^{2}}$) ones (**bottom**); from left to right, we show the pristine system followed by La, Eu, Gd and Yb adatoms on Gr/Ni(111). Red and blue are the majority and minority spin channels, respectively. The RE atomic electronic configuration is reported.

To better clarify this effect, in Figure 2, we report the surface-Ni and RE *d*-projected states. From the first row in Figure 2, it is evident that the hybidrization between the C-$p_{z}$ and Ni-$d_{z^{2}-r^{2}}$ orbitals underlies the spin-polarized state at the Fermi level, both in the pristine system and in the case of RE adsorption cases.

At the same time, from the second row in Figure 2, we note the presence of the RE-5$d_{xy,x^{2}-y^{2}}$ states hybridized with the C-$p_{z}$ (and Ni-$d_{z^{2}-r^{2}}$) ones only for La and Gd. Therefore, the spin-polarized electronic state at the Fermi level are extremely extended in real space: from the RE up to the surface-Ni layer in the out-of-plane direction.

**Figure 3:**
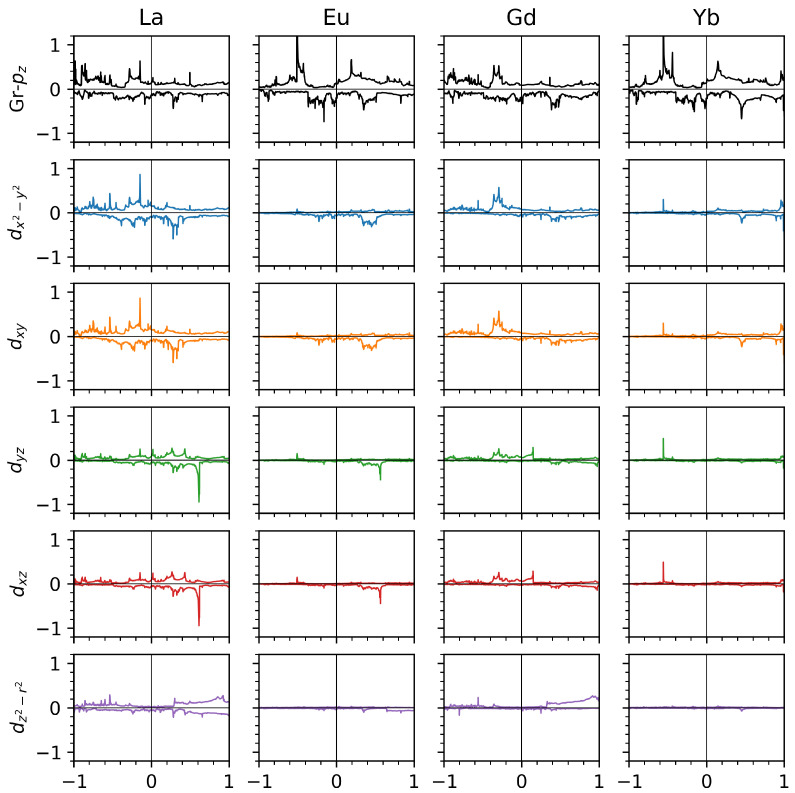
Selected orbital projected density of states (DOSs) for the studied systems. From top to bottom, we report the graphene $p_{z}$-projected density of states followed by the RE-d-projected DOSs. In the abscissa, we report the energies with respect to the $\varepsilon_{F}$ in eV. The DOS is shown in the ordinate, expressed in states/eV/spin: majority spin in the positive axis; minority spin in the negative one.

A counter-proof of the hybridization between the RE-$d_{xy,x^{2}-y^{2}}$ states with the C-$p_{z}$ orbitals comes from the analysis of the projected densities of states reported in Figure 3. As shown in the figure, we have a perfect superposition of the C-$p_{z}$ and RE-$d_{xy,x^{2}-y^{2}}$ states for the “up”-spin channel only for La and Gd adsorption. A negligible contribution from the other *d*-states is present.

To further expand on the investigation of this effect, we thus consider the last of Lanthanides (Lutetium) and the first of transition metals (Yttrium), having, respectively, a filled *f* orbital with a 5$d^{1}$ 6$s^{2}$ valence configuration (Lu) and a similar 4$d^{1}$ 5$s^{2}$ environment for Y (without *f*-states).

In agreement with the already observed behaviour, Lu and Y are also able to “detach” graphene from the Ni(111) surface, moving to a distance of 2.20 Å. A residual magnetization on the adatoms of 0.05 and 0.10 $\mu_{B}$, respectively, is present, ferromagnetically aligned with the Ni(111) substrate. The last Ni surface atoms exhibit a magnetization of 0.54 $\mu_{B}$, in both Lu and Y cases, in complete analogy with the previously considered RE-5*d* cases (La and Gd). The structural details for these last system are summarized in Table 2.

**Figure 4:**
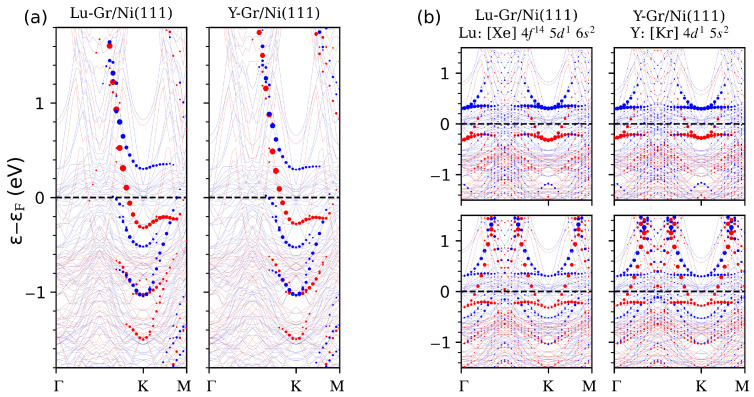
Panel (**a**): Unfolded electronic band structures projected on the carbon states (the size of circles is proportional to the spectral weight), for Lu (**left**) and Y (**right**) adatoms on Gr/Ni(111). (**b**): Band structures for the considered systems projected on the surface Ni($d_{z^{2}-r^{2}}$) states (**top**) and on the adatom ($d_{xy}+d_{x^{2}-y^{2}}$) ones (**bottom**), for Lu (**left**) and Y (**right**) cases. Red and blue are the majority and minority spin channels, respectively. The adatom electronic configuration is reported.

From Figure 4, the electronic properties of the systems are essentially indistinguishable between the Lu or Y adsorption cases. The adatom *d*-states interact with C-$p_{z}$ ones, destroying the minicone, in perfect agreement with what happens in the already considered cases of La and Gd adsorption. Thus, we conclude that the presence of *d*-states in valence is detrimental for the conservation of the graphene minicone.

## 4. Conclusions

In conclusion, this work confirms the possibility to induce, modify and dope the minicone state in graphene growth on Ni(111) using different kinds of adatoms, revealing the microscopic mechanisms that determine the hybridization between Ni-*d* states, graphene $p_{z}$ Dirac orbitals and RE valence electrons. Our predictions for Eu adsorption on graphene are in line with what was recently experimentally observed for Eu adatoms on a graphene/Co(0001) [33] system; the main difference was a reduced doping in graphene due to the Ni(111) substrate. Our extensive first-principles calculations reveal that the successful doping of the spin-polarized minicone is realized when the adatoms do not present *d*-states in valence, while their presence leads to the formation of a single-spin electron-like state crossing the Fermi level around the K-point of the graphene BZ, which flattens at ∼−0.25 eV along the K–M direction.

Our work proposes a feasible way to engineer the minicone band present in graphene on an Ni(111) substrate, which could serve as a material platform for applications in spintronics, transport experiments and Kondo physics [51,52].

## Figures and Tables

**Table 1:** Structural and magnetic properties of the studied systems: the average distance between graphene and the Ni(111) surface ($d_{C-Ni}$); the rippling of the graphene flake (Δ$d_{C}$); the average distance between the RE adatom and the carbon atoms ($d_{RE}$); the predicted atomic magnetic moments for the top-most Ni atoms ($m_{Ni^{*}}$) for the carbon atoms ($m_{C}$ for A and B sites, respectively) and for the RE atoms ($m_{RE}$).

	Gr/Ni(111)	La	Eu	Gd	Yb
$d_{C-Ni}$ (Å)	2.05	2.20	2.12	2.21	2.12
Δ$d_{C}$ (Å)	0.01	0.05	0.04	0.05	0.05
$d_{RE}$ (Å)	–	2.32	2.38	2.18	2.23
$m_{Ni^{*}}$ ($\mu_{B}$)	0.52	0.54	0.35	0.55	0.34
$m_{C}$ ($\mu_{B}$)	0.03/−0.01(5)	0.00	0.00/−0.00(7)	−0.00(6)/0.00	0.00/−0.00(8)
$m_{RE}$ ($\mu_{B}$)	–	0.16	−7.05	7.37	−0.00(8)

**Table 2:** Structural and magnetic properties of the studied systems. We report the average distance between graphene and the Ni(111) surface ($d_{C-Ni}$), the rippling of the graphene flake (Δ$d_{C}$) and the average distance between the RE adatom and the carbon atoms ($d_{RE}$). We also report the predicted atomic magnetic moments for the top-most Ni atoms ($m_{Ni^{*}}$), for the carbon atoms ($m_{C}$ for A and B sites, respectively) and for the RE atoms ($m_{RE}$).

	Lu-Gr/Ni(111)	Y-Gr/Ni(111)
$d_{C-Ni}$ (Å)	2.20	2.20
Δ$d_{C}$ (Å)	0.07	0.06
$d_{RE}$ (Å)	2.08	2.14
$m_{Ni^{*}}$ ($\mu_{B}$)	0.54	0.54
$m_{C}$ ($\mu_{B}$)	−0.00(2)/0.00(7)	0.00/0.00(5)
$m_{RE}$ ($\mu_{B}$)	0.05	0.10

## Data Availability

The data presented in this study are available from the corresponding authors upon reasonable requests.

## Abbreviations

The following abbreviations are used in this manuscript:

2D	Bi-Dimensional
RE	Rare Earth
FM	Ferromagnetic
BZ	Brillouin Zone
DFT	Density Functional Theory
DOSs	Density Of States
